# Supplementary material for: Predation and fragmentation portrayed in the statistical structure of prey time series
Source: BMC Ecol. 2009 May 6;9:10. doi: 10.1186/1472-6785-9-10 (PMC2689204; doi:10.1186/1472-6785-9-10)
Supplement: Additional file 2 — Voles and related classes ODDox Documentation. ODDox documentation of the agent-based model (ALMaSS) applied by Hendrichsen et al. The documentation is started by activating main.html. [file 1472-6785-9-10-S2.zip › Vole_ODDox/structrectangle.html]

ALMaSS ODDox: rectangle Struct Reference

- Main Page
- Related Pages
- Classes
- Files

- Alphabetical List
- Class List
- Class Hierarchy
- Class Members

# rectangle Struct Reference

`#include <PopulationManager.h>`

List of all members.

---

## Detailed Description

A struct defining two x,y coordinate sets.

|  |  |
| --- | --- |
|  |  |
| Public Attributes | |
| unsigned | m\_x1 |
| unsigned | m\_x2 |
| unsigned | m\_y1 |
| unsigned | m\_y2 |

---

## Member Data Documentation

unsigned rectangle::m\_x1

Referenced by Population\_Manager::Probe(), and Population\_Manager::ProbeFileInput().

unsigned rectangle::m\_x2

Referenced by Population\_Manager::Probe(), and Population\_Manager::ProbeFileInput().

unsigned rectangle::m\_y1

Referenced by Population\_Manager::Probe(), and Population\_Manager::ProbeFileInput().

unsigned rectangle::m\_y2

Referenced by Population\_Manager::Probe(), and Population\_Manager::ProbeFileInput().

---

The documentation for this struct was generated from the following file:

- PopulationManager.h

---

Generated on Thu Jan 22 14:13:46 2009 for ALMaSS ODDox by 
 1.5.6
